# Contribution of Solid Food to Achieve Individual Nutritional Requirement during a Continuous 438 km Mountain Ultramarathon in Female Athlete

**DOI:** 10.3390/ijerph18105153

**Published:** 2021-05-13

**Authors:** Kengo Ishihara, Naho Inamura, Asuka Tani, Daisuke Shima, Ai Kuramochi, Tsutomu Nonaka, Hiroshi Oneda, Yasuyuki Nakamura

**Affiliations:** 1 Department of Food Sciences and Human Nutrition, Faculty of Agriculture, Ryukoku University, Shiga 520-2194, Japan; n170316@mail.ryukoku.ac.jp (N.I.); tani@agr.ryukoku.ac.jp (A.T.); n20m005@mail.ryukoku.ac.jp (D.S.); n19m002@mail.ryukoku.ac.jp (A.K.); nakamura@belle.shiga-med.ac.jp (Y.N.); 2 Tail Ender’s Trail Running Life, Tokyo 176-0004, Japan; lacanmood2001@yahoo.co.jp; 3 Nagatasangyo Co., Ltd., Shiso 671-2544, Japan; oneda@nagatasangyo.co.jp; 4 Department of Public Health, Shiga University of Medical Science, Shiga 520-2192, Japan

**Keywords:** sports nutrition, continuous glucose monitoring, carbohydrate, protein, hydration, trail running, Freestyle Libre

## Abstract

Background: Races and competitions over 100 miles have recently increased. Limited information exists about the effect of multiday continuous endurance exercise on blood glucose control and appropriate intake of food and drink in a female athlete. The present study aimed to examine the variation of blood glucose control and its relationship with nutritional intake and running performance in a professional female athlete during a 155.7 h ultramarathon race with little sleep. Methods: We divided the mountain course of 438 km into 33 segments by timing gates and continuously monitored the participant’s glucose profile throughout the ultramarathon. The running speed in each segment was standardized to the scheduled required time-based on three trial runs. Concurrently, the accompanying runners recorded the participant’s food and drink intake. Nutrient, energy, and water intake were then calculated. Results: Throughout the ultramarathon of 155.7 h, including 16.0 h of rest and sleep, diurnal variation had almost disappeared with the overall increase in blood glucose levels (25–30 mg/dL) compared with that during resting (*p* < 0.0001). Plasma total protein and triglyceride levels were decreased after the ultramarathon. The intake of protein and fat directly or indirectly contributed to maintaining blood glucose levels and running speed as substrates for gluconeogenesis or as alternative sources of energy when the carbohydrate intake was at a lower recommended limit. The higher amounts of nutrient intakes from solid foods correlated with a higher running pace compared with those from liquids and gels to supply carbohydrates, protein, and fat. Conclusion: Carbohydrate, protein, and fat intake from solid foods contributed to maintaining a fast pace with a steady, mild rise in blood glucose levels compared with liquids and gels when female runner completed a multiday continuous ultramarathon with little sleep.

## 1. Introduction

Ultramarathon is a longer-distance marathon that has increasingly gained popularity in recent years [1]. The total energy expenditure of a 100-mile (160 km) ultramarathon reaches approximately 13,000 kcal in a 180 cm, 75 kg, middle-aged experienced male runner. The carbohydrate-derived energy in 140 min of the mountain marathon was reported to be 68%, which was lower than in 20 to 30 min of track (98%) or mountain running (86%). The percentage of lipid utilization would increase further as time and distance increased [2]. Thus, nutritional strategies are essential for ultramarathon runners wanting to improve their race results and also for those focusing primarily on finishing the event.

For endurance sports, the recommended carbohydrate intake is 30–60 g/h; however, for exercises lasting more than 3 h, the advocated recommendation is higher (i.e., ≤90 g/h and glucose:fructose ratio of 2:1) [3,4]. For a single-stage ultramarathon that generally lasts for more than 3 h, a carbohydrate level of 30–50 g/h is recommended because of numerous barriers to achieve 90 g/h consumption of a multiple-transportable carbohydrate blend [5]. Some of these barriers were described as follows. First, observational studies demonstrated that the actual carbohydrate intake during ultramarathons was less than 60 g/h in most runners [2,6,7], including slower runners consuming 37 g/h [8], and very few reached more than 60 g/h [9,10]. Second, the absolute exercise intensity of an ultramarathon was not as high as some other endurance activities because of its extremely long duration (6, 13, 24, 48, 72, or 10 days) [11]. Third, intestinal absorption might be affected by undertaking highly intensive and long-duration exercises because of the changes in the splanchnic blood flow. In addition, heat, endotoxin, or vertical shaking of their digestive system during rough terrain races could lose the appetite of ultramarathon runners [11,12]. Fourth, ultramarathon runners have to carry their food and fluids in their backpacks during long hours of racing; considering the additional weight being carried, the exercise intensity was increased [8]. Fifth, runners might find food intake difficult while maintaining their balance with both hands when running down steep mountains or climbing steep slopes.

In recent years, races and competitions of over 100 miles have increased, but the specific nutritional and hydration requirements during a continuous multiday ultra-endurance running are still insufficiently known. Reports on races longer than 100 miles [13,14,15,16,17,18] and especially on the nutritional intake are also limited [19,20,21].

A review on nutritional supplementation during ultramarathons mainly covers running events of 100–160 km, with a maximum of 217 km [2]. Likewise, a recently published position statement of the International Society of Sports Nutrition [5] and practical recommendations for ultramarathon events [8,14,22] are mainly based on 100–160 km studies.

Since energy intake in an ultramarathon usually exceeds the energy expenditure [5], the effects of energy deficiency would be apparent when the competition time becomes longer than several days and close to a week. The energy deficit could lead to hypoglycemia, degradation of organs, reduction of energy substrates in the blood, and decline in running performance. Firstly, hypoglycemia could reduce the running speed [23]. Hence, the minimum required amount of carbohydrates in each athlete must be identified to maintain their blood glucose levels using a continuous glucose monitoring system. Continuous blood glucose monitoring has widely been used in patients with diabetes and healthy people during exercise [24,25].

Secondly, degradation of tissues could become apparent [8,26,27]. Degradation of tissues could be attenuated by carbohydrate supplementation of 120 g/h, which is far above the recommended amount. However, it cannot enhance running performance and can even increase the incidence of gastrointestinal problems [28,29].

Third, the effects of sleep deprivation on metabolism and athletic performance might be more pronounced. The effects of sleep loss on physiological responses and exercise remain equivocal. An exhaustive review reported that sleep deprivation decreased exercise time to exhaustion, mean power, and increased heart rate [30]. In healthy non-athletes, sleep restriction induced both weight gain and diabetes risk by altering the glucose metabolism, upregulation of appetite, and decreased energy expenditure [31]. However, there are no reports on sleep deprivation and glucose control during ultra-distance events.

Optimal nutrition leads to a decreased risk of energy depletion, better performance [32], the prevention of acute cognitive decline, and improved athlete safety on ultramarathon courses with technical terrain or those requiring navigation [5]. However, the execution of the precise nutrition plan might be difficult for the runner [33] because the nutrient requirements for ultramarathon racing vary greatly, depending on the individual [5], such as age, sex, body mass, or exercise intensity.

A continuous glucose monitoring system encodes individual fluctuations of blood glucose levels in all life situations, including extreme endurance sports [24,25,34,35,36]. For endurance athletes, wearable devices enable them to compete while maintaining social distance and race across time and space by keeping the records in the cloud [37]. A combination of these devices could potentially improve our understanding of the complex interplay between nutrition and exercise performance.

Through analyzing the relationship between blood glucose fluctuation, running speed, and nutrient intake, one’s optimal nutritional requirement could be determined. This approach might help acquire one’s appropriate energy and nutrient intake, especially during long-distance events [24,34,35,36].

This study aimed to examine the variation of blood glucose control and its relationship with the nutritional intake and running performance in a professional female athlete during the 155.7 h of 438 km ultramarathon race with little sleep.

## 2. Materials and Methods

### 2.1. Study Design

This case study was designed to define the normal range of blood glucose during a continuous multiday ultramarathon with little sleep and observe the relationships among nutrient intakes, running performance, and blood glucose level. The Ryukoku University Human Research Ethics Review Board (No. 2019-35) approved all our procedures, which complied with the code of ethics of the World Medical Association (Declaration of Helsinki). Written informed consent was obtained from the participant before the study commenced.

### 2.2. Study Population

A professional female trail runner (age, 44 years; height, 1.52 m; body mass, 42 kg; body mass index, 18.2 kg/m^2^, body fat percentage 18%) voluntarily participated in the study. Her annual mileage, elevation gain, and training time were 4440 km, 212,000 m, and 720 h, respectively. She had completed various ultramarathon races, including 330 km certified by the International Trail Running Association, in the last 3 years. In the 35 international races she has competed in, she finished 29 times in the top 10, 16 times in the top 3, and won 4 times. Hence, she is well experienced in ultramarathons.

### 2.3. Mountain Ultramarathon Course

This study was conducted during a mountain ultramarathon challenge for the fastest-known time in Shiga Round Trail/Shigaichi (https://fastestknowntime.com/route/shiga-round-trail-shiga-ichi-japan, accessed on 12 May 2021) held around the Lake Biwa, the largest lake in Japan (ambient temperature range: 18.3 °C–30.5 °C, relative humidity range: 42–75%), during the first week of June 2020. The distance of the course covered 438 km, and the total elevation gain was 28,300 m. Over 90% of its length unfolded along the unpaved forestry trail. The course was divided into 32 segments by 33 timing gates where investigators recorded each runner’s passing time. The distances between each timing gate were 13.69 ± 5.3 SD km (range: 5.9–26.8 km). The running time and speed between each timing gate were obtained by investigators, and the global positioning system (GPS) was recorded by a wristwatch (SUUNTO 9, Suunto, Finland). Furthermore, the location and running speed of the runner were broadcasted live on the internet via a GPS-based tracking system (https://ibuki.run/, archived on 12 May 2021, IBUKI live, ONDO Inc., Kyoto, Japan). Specifically, the running time between each timing gate was 4:52 ± 2:16 h (range: 1:10–11:40 h). During the race, the runner carried her backpack containing necessities, such as food and fluid, which could be replenished at each timing gate. The total running time was 155.7 h, including 16.0 h of rest and sleep. The total hours of rest and sleep each day were 0:20, 1:55, 1:40, 4:00, 3:30, 2:45, 1:50 h:min plus 2 h of fragments of sleep during running.

### 2.4. Running Pace Data Collection and Standardization

The running performance during the ultramarathon was calculated using the following arbitrary formula:

Pace (min/km) = (E − A)/(Distance of the segment (km))

where

E: the estimated running time of the segment (min);
A: the actual running time of the segment (min).

The pace value was positive when the runner ran faster than the estimated running time but negative when the runner ran slower. Before the ultramarathon, the runner ran 3 1/2 trial laps of this course to determine the estimated running time of the segment.

### 2.5. Glucose Data Collection and Standardization

All of the blood glucose profile was monitored by flash glucose monitoring (FGM), a minimally invasive method described in previous reports [38,39,40,41,42]. Briefly, the FGM system (FreeStyle Libre; Abbott Diabetes Care, Alameda, CA, USA) mechanically reads and continuously measures the glucose concentration in the interstitial fluid collected right below the skin and subsequently reveals the corresponding ambulatory glucose profile. From one day before the race to three days after the race, the FGM sensor was applied at the back of the runner’s upper arm, and glucose concentrations were obtained every 15 min [39]. The highest and lowest glucose concentration levels in each segment and their difference were used as representative values in every segment.

The samples for the plasma clinical parameters were collected after an overnight fast one month before and one week of an off-training period after the ultramarathon and analyzed. The ultramarathon was planned to take place a month earlier, but due to the COVID-19 infection situation and the soft lockdown by the Japanese government, the start of the ultramarathon was delayed by a month. Blood was collected in a clotted vial, and the serum obtained was analyzed by clinical laboratory testing (Falco Biosystems, Inc., Kyoto, Japan). Aspartate aminotransferase (AST), alanine aminotransferase (ALT), and creatine kinase (CK) were estimated by the JSCC standard method. Alkaline phosphatase (ALP) and lactate dehydrogenase (LDH) were estimated by the IFCC standard method. Triglyceride and LDL-cholesterol were estimated by the enzyme colorimetric method. Sodium, potassium, and chloride were estimated by the ion-selective electrode method. Calcium was estimated by the arcenazo III colorimetric test.

### 2.6. Diet Supply Data Collection

Investigators followed the runner and recorded the entire food and drink intake in relays throughout the ultramarathon. They reported the timing and volume of consumed food and fluid products based on pictures taken during the race. In detail, one or two of the investigators always ran with the runner, taking turns in each segment. They checked the current location, based on GPS, when the runner consumed the refueling meal and recorded the consumption point on a map. Food and fluid products consumed more than 60 min before the start of the ultramarathon were excluded in the nutrient intake calculation with reference to previous studies [23,43]. The nutrition information indicated on the cover of the food and fluid products was our basis when calculating the energy and nutrient intake. If data were unavailable, the intake was calculated according to the standard tables of food composition in Japan 2015 (7th revised edition) [44]. The energy and nutrient intake was expressed relative to the pre-race body weight (kg) per running time (h). In reference to previous research [23,44], all foods were categorized as follows: sports drinks (isotonic and hypertonic formulas), cola, gels, milk product, tea, soup, other liquids (all other drinks consumed), fruits, sweets, bars, noodles, bread, rice products, wheat products, powder, and other solids (all other products consumed).

As shown in Table 1, the runner consumed energy and nutrients from liquids, gels, fruits, sweets, and other solids. The hourly intake of energy, protein, fat, carbohydrate, water, and salt was 170.8 kcal, 5.9 g, 3.1 g, 29.7 g, 263.0 g, and 1.1 g, respectively. The protein:fat:carbohydrate ratio of the ingested nutrients was 13.7:16.5:69.5. The runner consumed 30.1% and 58.3% of their energy from liquids and gels, and solids, respectively. Identically, the intake of carbohydrates from solids (44.4%) was similar to that from liquids or gels (41.4%). Meanwhile, proteins (89.5%) and fats (84.3%) were mostly consumed from solids. Other solids included smoked chicken, potatoes, risotto, lasagna, and protein powder. Other liquids included smoothies and coffee.

### 2.7. Statistics

Herein, numerical data are presented as means and standard deviations unless otherwise specified. Data from a female ultrarunner were processed and analyzed in GraphPad Prism for Mac (version 9.0.1, GraphPad Inc., San Diego, CA, USA). The associations between the running performance, glucose level, and nutrient intake were investigated using Spearman’s rank correlation coefficients. The differences among each situation of the blood glucose level were compared by a Mann–Whitney test by ANOVA followed by Dunn’s multiple comparison test for the comparison among more than three groups. Results were considered significant when *p* < 0.05. Limitations of the single-subject research design are the generalizability of the study conclusions and were described in the discussion section.

## 3. Results

### 3.1. General Results of Blood Glucose Fluctuation during the Ultramarathon

**Figure 1:**
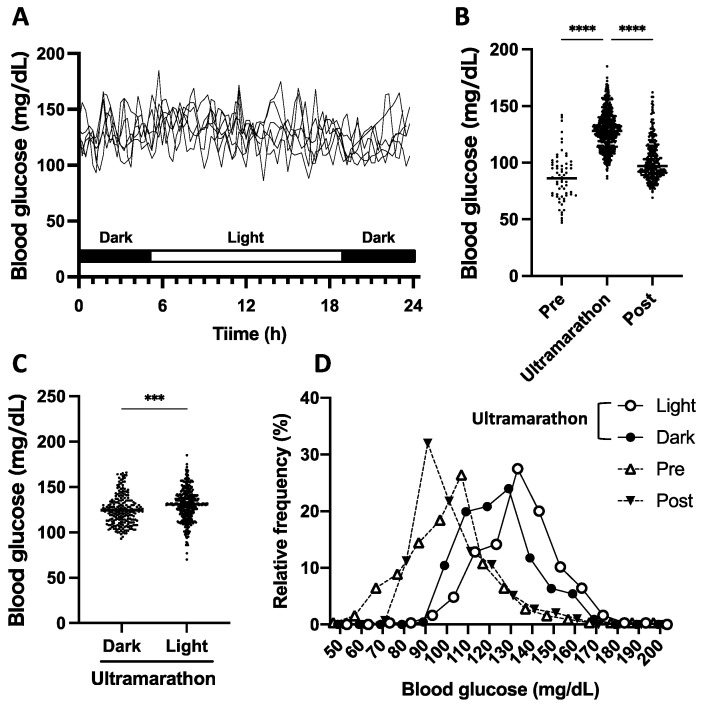
Blood glucose fluctuation during the 7-day ultramarathon. Each solid line represents the daily glucose variation (**A**). Scatter plot of blood glucose during a preliminary, ultramarathon, and post-ultramarathon periods, respectively (**B**). Scatter plot of blood glucose levels during night (dark) and day (light) throughout the ultramarathon (**C**). Histogram of blood glucose fluctuation during preliminary, ultramarathon (night and day), and post-ultramarathon periods (**D**). *** *p* < 0.001, **** *p* < 0.0001 The differences between dark and light were compared by the Mann–Whitney test, and those among pre, ultramarathon post were compared by the Kruskal–Wallis nonparametric ANOVA, followed by Dunn’s multiple comparison test.

During the 7-day ultramarathon, the regular circadian rhythms, including breakfast, lunch, and dinner, almost disappeared, as detected in the blood glucose levels (Figure 1A). Additionally, the mean blood glucose levels (25–30 mg/dL) were higher than those during the preliminary and post-ultramarathon periods (*p* < 0.0001, Figure 1B). The mean daytime and nighttime blood glucose levels during the ultramarathon were 130.0 ± 16.2 and 124.7 ± 17.3 mg/dL, respectively, with a slight difference (*p* < 0.001, Figure 1C). Moreover, the blood glucose levels during the ultramarathon were controlled within a narrower range than during the preliminary period (Figure 1D).

### 3.2. Relationship between the Amount of Nutrient Intake and Maintenance of Glucose Level during the Ultramarathon

**Figure 2:**
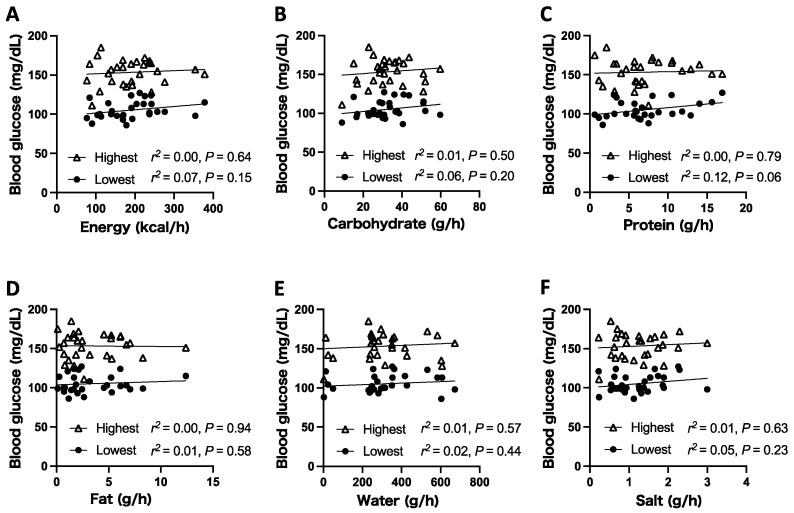
Scatter plots showing the relationships between nutrient intake and blood glucose level. The intake of energy (**A**), carbohydrate (**B**), protein (**C**), fat (**D**), water (**E**), and salt (**F**) was calculated according to the consumed food and fluid products. Each plot indicates one segment.

Significant correlations between the runner’s blood glucose levels and nutrient intake were not observed. The lowest glucose level between the segments tended to correlate with protein intake (*p* = 0.06). The highest blood glucose level in the segment was not significantly related to nutrient intake. The difference between the two lines was 50 mg/dL approximately, which was the difference between the highest and lowest blood glucose levels in each segment. Among all nutrients, the difference did not vary significantly from about 50 mg/dL, regardless of the amount consumed (Figure 2).

### 3.3. Relationship between Glucose Level and Running Pace

**Figure 3:**
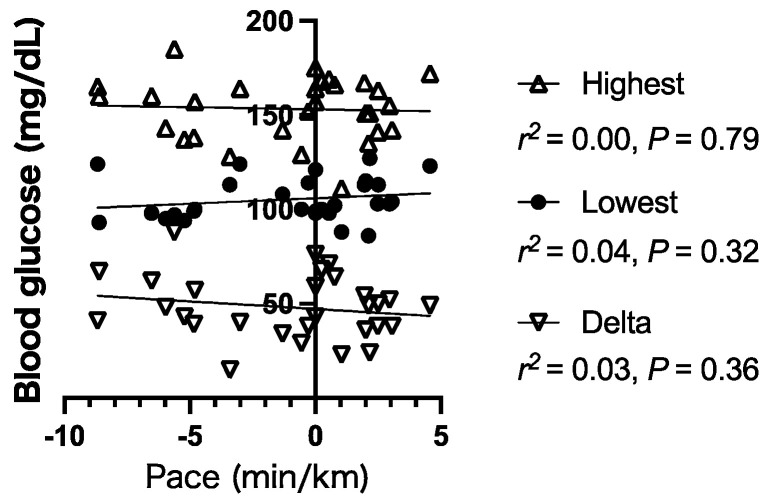
Relationship between the running pace and the highest blood glucose level, lowest blood glucose level, and delta (difference between the highest and lowest blood glucose levels) between segments. The pace was calculated from the difference between the estimated running time and actual running time of the segment, as described in the Methods section. Briefly, the pace value was positive when the runner ran faster than the estimated running time and negative when the runner ran slower.

During exercise, the runner was within the expected normoglycemic range (86–185 mg/dL), with no extreme hyperglycemia or hypoglycemia (Figure 1). Therefore, the running pace had no significant correlation with the highest blood glucose level (*p* = 0.79), lowest blood glucose level (*p* = 0.32), and delta (difference between the highest and lowest blood glucose levels, *p* = 0.36) between segments (Figure 3).

### 3.4. Relationship between the Amount of Nutrient Intake and Running Pace during the Ultramarathon

**Figure 4:**
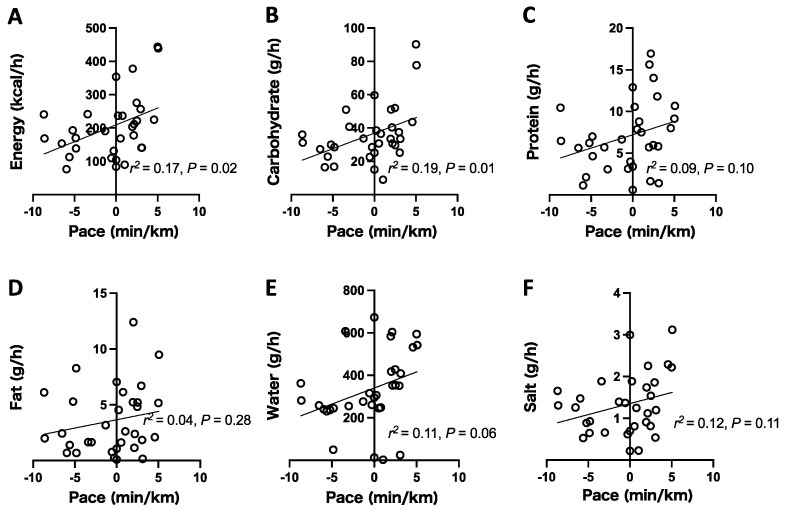
Scatter plots showing the relationships between nutrient intake and running pace. The intake of energy (**A**), carbohydrate (**B**), protein (**C**), fat (**D**), water (**E**), and salt (**F**) was calculated according to the nutrition information of the consumed food and fluid products. Each plot indicates one segment.

The running pace significantly correlated with energy (*p* = 0.02) and carbohydrates (*p* = 0.01). The running pace tended to correlate with protein (*p* = 0.10) and water intake (*p* = 0.06). Other nutrient intake data did not show the correlation with the running pace (Figure 4).

### 3.5. Comparison of Nutrient Intake between Fast and Slow Running Paces

**Figure 5:**
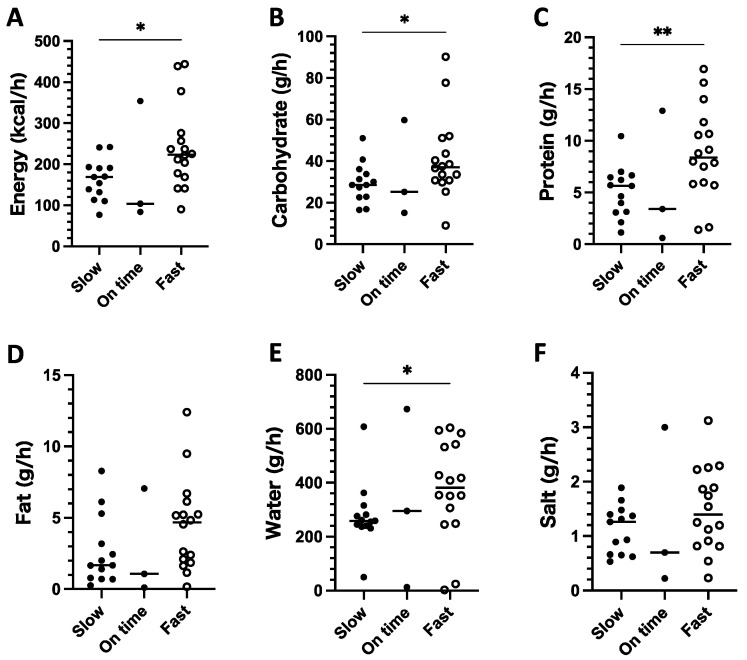
Comparison of energy (**A**), carbohydrate (**B**), protein (**C**), fat (**D**), water (**E**), and salt (**F**) intake among different running paces (fast, when the running pace was faster than planned; on time, when the running pace was the same as planned; slow, when the running pace was slower than planned). * *p* < 0.05 and ** *p* < 0.01 between fast and slow, Mann–Whitney test. The horizontal bar represents the median in each group.

The energy and nutrient intake in the positive running pace (faster than planned) were compared with that in the negative running pace (slower than planned). The energy (*p* < 0.01), carbohydrate (*p* < 0.05), protein (*p* < 0.01), fat (*p* < 0.05), water (*p* < 0.01), and salt (*p* < 0.05) intake in the positive running pace was significantly higher than that in the negative running pace. In the positive running pace, the median hourly nutrient intake for carbohydrate, protein, fat, water, and salt was 38.0 g/h, 9.0 g/h, 5.0 g/h, 413.0 mL/h, and 1.6 g/h, respectively, and the median hourly energy intake was 225 kcal/h (Figure 5).

### 3.6. Comparison of Food Type between Fast and Slow Running Paces

**Figure 6:**
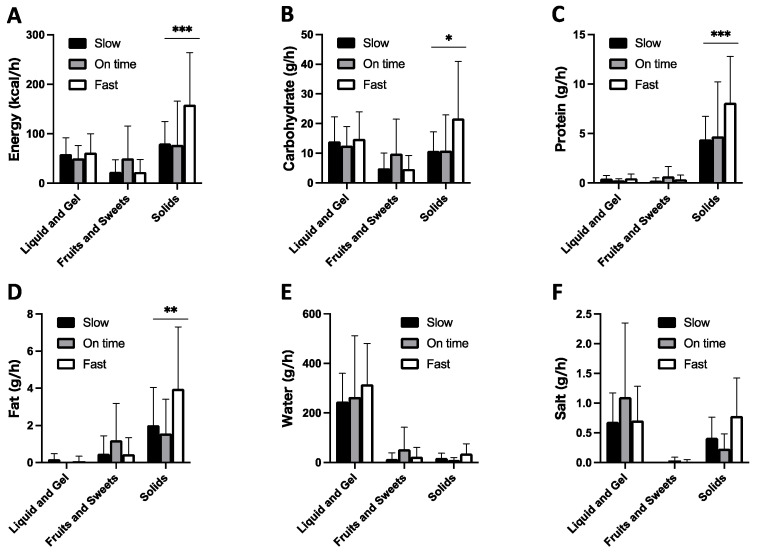
Comparison of product type for energy (**A**), carbohydrate (**B**), protein (**C**), fat (**D**), water (**E**), and salt (**F**) consumption among different running paces (fast, when the running pace was faster than planned; on time, when the running pace was the same as planned; slow, when the running pace was slower than planned). * *p* < 0.05, ** *p* < 0.01, *** *p* < 0.001 between fast and slow, Mann–Whitney test. Values are means ± SEM.

Food product types used for energy and nutrient consumption were compared in terms of the positivity or negativity of the running pace. When the running pace was positive, the energy and nutrient intake from solids was approximately two times higher than that when it was negative (*p* < 0.05). The water intake was mainly derived from liquids or gels and the intake of liquid (Figure 6).

### 3.7. Change of Serum Parameters Pre- and Post-Ultramarathon

Triglycerides in the blood were greatly reduced, suggesting that lipid utilization contributed significantly to energy production. The total protein in the blood was also slightly decreased. The decrease in ALP could be due to insufficient zinc intake. On the other hand, there was only a slight increase in AST and ALT compared to the marked increase in LDH and CK, suggesting that although there was muscle damage, the damage on the liver function was small (Table 2).

## 4. Discussion

This study aimed to examine the variation of blood glucose control and its relationship with nutritional intake and running performance in a professional female athlete during the continuous over 400 km ultramarathon race with little sleep. Diurnal variation had almost disappeared with the overall average glucose increase of approximately 30 mg/dL compared to resting. A significantly faster running speed correlated with a higher energy and nutrient intake from solid foods than from gels and liquids. Interestingly, the median energy and carbohydrate intake in the fast-running pace were within the recommended energy and carbohydrate intake, mainly covered 100–160 km ultramarathon [5,6].

Protein intake contributed to the maintenance of blood glucose levels as carbohydrate intake was at the lower end of the recommended amount. Sufficient energy and nutrient intake prevented hypoglycemia, thereby maintaining the running speed during the ultramarathon. Consistent with the findings from a 100-mile race [23], the highest blood glucose concentration obtained was not associated with the running speed, indicating that instead of the rapid availability of carbohydrates, nutrient intake from solid foods for controlling glucose homeostasis was the key determinant of performance especially in an ultramarathon of over 400 km.

Recording the amount of energy and nutrient intake during prolonged exercise events had corresponding difficulties. A bicycle equipped with a camera cycled alongside the runner to accurately record the results [45]. In the present study, given that the course mostly covered a single track where bicycles could not pass, the ultramarathon runners were followed by other runners to record the food and drink intake; hence, the energy and nutrient intake recorded was precise and valuable. This ultramarathon gained considerable attention that several runners took turns to accompany the ultramarathon runner for approximately one week to keep track of her meals and drinks.

Gluconeogenesis played an essential role in maintaining blood glucose levels during an ultramarathon, considering that meeting carbohydrate consumption throughout the entire ultramarathon race was not feasible, not even in typical durations of an ultramarathon (6–48 h). Energy deficiency was common in ultramarathons [5,6,7,32,45,46,47,48]. Studies using a doubly labeled water technique or respiratory gas analysis estimated that the energy expenditure during 160 km ultramarathons was approximately 13,000 kcal [2,49,50]. The previously reported rates of gluconeogenesis and hepatic glycogenolysis in a resting state in low-carbohydrate–fed subjects were 0.07 and 0.03 g/kg/h, respectively [51]. The sum of these two values (0.1 g/kg/h), otherwise known as endogenous glucose production, would be the minimum required amount of carbohydrates to maintain blood glucose levels during a resting state. The endogenous glucose production significantly increased to 0.36 g/kg/h during exercise at 55% of peak power output [51] or 0.48 g/kg/h during exercise at the lactate threshold level in fasted, well-trained subjects [52]. In accordance with this calculation, the runner in the present study required 29.7 and 5.9 g/h of carbohydrate and protein intake, respectively, to maintain her blood glucose concentrations during the ultramarathon.

Consuming mostly solid foods alongside other carbohydrate forms can be a practical option for the supplementation of adequate energy and nutrients with fewer gastrointestinal problems, especially for a race that lasts for several days. Previous reports using ^13^C-labeled isotopes revealed that carbohydrates from solid foods (as well as from liquids) were effectively oxidized during exercise and could suppress gastric emptying compared with the liquid form [43]. Solid foods slightly elevated blood glucose levels and secreted less insulin [53] and glucose-dependent insulinotropic peptide compared with liquid foods [54]. Furthermore, gastric emptying of semisolid food was not affected by exercises at intensities of the 40% VO_2_ peak [55]. Solid foods could maintain the same blood glucose levels as gel foods and perform the same intensity of cycle exercise and time trials [56]. According to recent reports, ingestion of a larger volume of carbohydrate solution at less frequent intervals during prolonged submaximal running spared endogenous carbohydrate oxidation rates. It did not cause increased markers of gastrointestinal discomfort compared with the smaller volumes at more frequent intervals [57].

A marked increase in CK, compared with the other biochemical markers, such as ALT, AST, LDH, were reported in previous studies on 130 to 160 km of ultramarathon [26,27]. In a longer-distance ultramarathon, a significant increase in CK was observed [8]. On the other hand, ALP was mildly elevated in this previous study but decreased in the present study. The reduction of ALP might be due to insufficient zinc intake in this study.

In the present study, there was a correlation between nutrient intake and speed, as the intake of energy and nutrients were insufficient compared with the energy expenditure during the ultramarathon. Insufficient intake was speculated by the decrease in blood triglycerides and total protein concentration in the present ultramarathon. Our previous study also revealed that the lowest blood glucose level in each section was the cause of the running speed reduction, though the highest blood glucose level in each section of the run was not related to the running speed [23]. Fatigue is caused by various factors, and excessive intake did not entirely enhance performance. The weak correlation between the blood glucose level and the running speed could be explained by the previously reported gender-specific differences in fuel utilization during exercise. Females showed higher lipid oxidation caused by higher plasma adiponectin levels [58], higher muscle triglyceride utilization [59], low plasma glucose levels [60], and higher fasting hepatic glucose uptake than males [61].

Levels of glucose increased by an average of approximately 20 percent during the early part of nocturnal sleep but returned to baseline levels in the morning because of reduced glucose utilization during sleep [62]. Similarly, glucose tolerance was optimal in the morning and reached its minimum in the middle of the night [63,64]. Another study revealed an association between sleep and glucose regulation during constant glucose infusion, which was a condition that inhibited endogenous glucose production and, therefore, revealed changes in glucose utilization [62].

The intravenous glucose tolerance test during the sleep restriction condition demonstrated that the rate of glucose clearance was approximately 40% lower and the acute insulin response to glucose was 30% lower compared to the sleep extension condition [65]. Interestingly, the diurnal rhythm of blood glucose levels in the present study was almost abolished compared to the resting state, and the difference between daytime and nighttime blood glucose levels was significant but not pronounced. This change was caused by the combined factors of running and sleep deprivation, but the mean and SD of blood glucose levels did not gradually increase along with the accumulation of sleep deprivation during the ultramarathon. These results suggested that running in a sleep-deprived state for at least up to a week did not cause extreme fluctuations in blood glucose levels.

Sleep deprivation of 30 to 72 h did not drastically affect cardiovascular and respiratory responses to exercise of varying intensity or the aerobic and anaerobic performance capability [66]. For example, during prolonged treadmill walking at about 80% of the VO_2max_, the reduction of work time to exhaustion was only 11% after 30 h of sleep deprivation [67]. Another study reported that the maximal isometric and isokinetic muscular strength and endurance of selected upper and lower body muscle groups, the performance of the Wingate Anaerobic Power Test, simple reaction time, the blood lactate response to cycle exercise at 70% VO_2max_, and most of the cardiovascular and respiratory responses to treadmill running at 70% and 80% VO_2max_, were not significantly altered as a result of sleep deprivation of 60 h [68]. Although the sleep deprivation in this study was 155.7 h, the runner was not in complete sleep deprivation, and its relationship to performance needed to be carefully examined.

Meanwhile, the main limitation of this study was the number of subjects. It was not feasible to have sufficient subjects, as few people even attempt to run through the entire route of the 438 km of a mountain ultramarathon for continuous several days. In the present ultramarathon, another runner started simultaneously but retired in the middle of the race. Though this research was a case study, our study athlete set the fastest time; thus, our record could be considered valuable. Our observational study supported the effectiveness of the position statement of the International Society of Sports Nutrition [5] and practical recommendation for ultramarathon participants to prevent hypoglycemia during exercise.

The limitations of the single-subject research design were the generalizability of the study conclusions and the methodological and statistical assumptions that were typically needed for inferential statistical tests. A single-subject design provided limited support for conclusions regarding populations of subjects [69]. Nonetheless, single-subject studies have been conducted in rehabilitation, disability [70,71], or psychological research [72]. These study designs employ a comparison in the AB design. In this design, A represents the baseline, and B represents the treatment. The subject is treated repeatedly as AB or ABA [73,74]. Similarly, ABAB or other extensions of AB designs are stronger designs than the simple phase change of an AB design [71]. In the present study, the statistical data was processed as an extended AB design consisting of segments of faster or slower than the planned pace. In the future, these findings need to be accumulated to reach a general conclusion.

Another methodological limitation was the large fluctuations in running speed during the ultramarathon. The running speed in the mountain ultramarathon usually varied by vast diversities of terrain [75] and physiological changes such as muscle fatigue and energy deficiency. We could not measure the heart rate as the runners did not tolerate the discomfort of wearing the belt for six days. We used a GPS tracking system to monitor this run, but it was difficult to calculate the intensity from the distance and slope because more than 90% of this route was a track in the forest, and the surface was diverse. Therefore, the running speed values of the runner were standardized using the preliminary planned running time, which was obtained by allowing the runner to run 3 1/2 trial laps of this course before the ultramarathon. The precise and objective power meters were already applicable in cycling studies [76].

Continuous glucose monitoring systems were less accurate than the gold standard for intermittent self-measurement of blood glucose [40]. The present research adopted the system, which was pronounced as superior performance during exercise compared with the other GM systems [41]. Although we rarely observed hypoglycemia in this study, caution should be exercised regarding the accuracy of values at low blood glucose levels as the median absolute relative difference between the reference values and those obtained by the sensor across the glycemic range overall was 22 (13.9–29.7)% and was 36.3 (24.2–45.2)% during hypoglycemia, 22.8 (14.6–30.6)% during euglycemia and 15.4 (9–21)% during hyperglycemia [77].

GPS monitoring throughout the entire running and sharing the runner’s location was linked to the assured dietary support and safety of the runner in the present study. Sharing the blood glucose level as well was expected to ensure further safety. Accurate physical workload calculation based on GPS monitoring or running power meter would enable a more accurate analysis of the running performance and blood glucose fluctuations.

## 5. Conclusions

In conclusion, the diurnal variation of the plasma glucose level had almost disappeared with the overall slight glucose increase during a continuous multiday ultramarathon in a female athlete. The intake of protein and fat directly or indirectly contributed to maintaining the blood glucose levels and running speed as gluconeogenesis source or energy source when the intake of carbohydrates was at the lower limit of dietary recommendation. Carbohydrate, protein, and fat intake from solid foods contributed to maintaining a fast pace compared with liquids and gels.

## Figures and Tables

**Table 1:** Total energy and nutrients consumed during the ultramarathon.

	Energy, kcal	Protein, g	Fat, g	Carbohydrate, g	Water, g	Salt, g
**Liquids and gels**	**7999**	**56**	**17**	**1911**	**35,018**	**84**
Sports drink	1467	5	0	377	6617	13
Cola	87	0	0	21	103	0
Gel	1255	9	0	306	208	44
Milk product	3324	32	6	772	11,889	8
Tea	1120	0	0	284	14,216	14
Soup	331	8	11	48	683	5
Other liquids	415	2	0	103	1302	0
**Fruits and sweets**	**3075**	**37**	**59**	**657**	**2329**	**1**
Fruit	1122	14	3	291	1601	0
Sweet	1953	23	56	366	728	1
**Solids**	**15,516**	**815**	**409**	**2047**	**3579**	**77**
Bar	3120	260	91	312	78	8
Noodle	1678	53	29	287	1069	17
Rice product	5334	125	91	965	1050	32
Wheat product	2164	74	88	250	463	7
Powder	651	119	7	22	12	2
Other solids	2569	184	103	211	907	11
**Total**	**26,590**	**908**	**485**	**4615**	**40,926**	**162**
**Average per hour**	**171**	**5.9**	**3.1**	**29.7**	**263**	**1.1**

The subtotal of each category is shown in bold.

**Table 2:** Serum parameters pre- and post-ultramarathon compared with the Japanese population.

		Normal Range	Ultramarathon	
		Mean (95% CI)	Pre	Post
Total protein	g/dL	7.4 ± 0.5 (6.5–8.3)	6.9	5.8 ^L^
Triglyceride	mg/dL	89.5 ± 30.4 (30.0–149.0)	140	33
LDL-cholesterol	mg/dL	104.5 ± 17.6 (70.0–139.0)	65 ^L^	42 ^L^
AST	U/L	23.0 ± 7.7 (8.0–38.0)	31	87 ^H^
ALT	U/L	23.5 ± 9.9 (4.0–43.0)	35	84 ^H^
ALP	U/L	232.0 ± 62.2 (110.0–354.0)	258	70 ^L^
LDH	U/L	183.0 ± 31.6 (121.0–245.0)	184	428 ^H^
CK	U/L	117.0 ± 40.3 (38.0–196.0)	102	1312 ^H^
Na	mEq/L	142.5 ± 3.8 (135.0–150.0)	137	142
Cl	mEq/L	104.0 ± 3.1 (98.0–110.0)	101	106
K	mEq/L	4.4 ± 0.5 (3.5–5.3)	4.7	3.9
Ca	mg/dL	9.3 ± 0.5 (8.4–10.2)	9	8.5

^L^, lower than normal range CI; ^H^, higher than normal range; AST, aspartate aminotransferase; ALT, alanine aminotransferase; ALP, alkaline phosphatase; lactate dehydrogenase; LDH, lactate dehydrogenase; CK, creatine kinase.
